# Evaluating Treatment Outcomes and Tuberculosis Infection Risks: A Comparative Study of Centralized Hospitalization vs. Home-Based Treatment

**DOI:** 10.3390/tropicalmed9050119

**Published:** 2024-05-18

**Authors:** Fangming Xianyu, Yuemei Huang, Shengqiong Guo, Virasakdi Chongsuvivatwong

**Affiliations:** 1 Department of Epidemiology, Faculty of Medicine, Prince of Songkla University, Hat Yai 90110, Thailand; a992584760@gmail.com; 2 Guizhou Medical University, Guiyang 550031, China; huangyuemeiii@gmail.com; 3 Guizhou Provincial Center for Disease Control and Prevention, Guiyang 550004, China

**Keywords:** pulmonary tuberculosis, treatment outcome, centralized hospitalization

## Abstract

**Background:** Guizhou Province in Southwest China has experimented with a centralized hospitalization (CH) treatment for active and severe cases of pulmonary tuberculosis (PTB). The objective of this study was to compare treatment outcomes of patients with tuberculosis (TB) receiving care in a CH setting with those receiving home-based (HB) care. In addition, this study aimed to assess the probability of their household contacts contracting tuberculosis infection. **Method:** A retrospective review of medical records was undertaken for patients with TB who completed their treatment in four counties in Guizhou, China, spanning from January 2022 to August 2023. In addition, a cross-sectional survey was conducted on the tuberculin skin test (TST) among household contacts of new patients with TB who had completed their treatment. **Results:** In the retrospective study, 94.8% had successful CH treatment, and 93.1% had successful HB treatment (*p* value = 0.70). In the prospective study, 559 and 448 household contacts of patients receiving CH treatment had 16 positive and 89 negative TST results, whereas those with HB treatment showed 26 positive and 74 negative TST results. Regarding a logistic regression analysis, the CH group was nearly two times more likely to test negative on the TST, 1.95 (95% CI: 0.98, 3.92). After adjusting for confounding variables, the odds ratio increased significantly to 4.42 (95% CI: 1.22, 16.04). **Conclusions:** CH for treatment of TB did not show superior success rates, but it may reduce the risk of transmitting tuberculosis infection to household contacts compared to home treatment.

## 1. Background

Tuberculosis (TB) is a disease caused by mycobacterium tuberculosis (MTB) [1]. TB is not only among the top 10 causes of death globally, but it also causes the absolute number of deaths due to an infectious disease [2]. Three national TB prevalence surveys conducted in the Chinese mainland showed that the incidence of TB has decreased by over 50% in the past 20 years due to the widespread adoption of the Directly Observed Treatment Short-course (DOTS) and End TB strategies [3,4].

Tuberculosis infection is defined as the presence of MTB infection in which the bacteria are alive but are not currently causing active TB disease [5]. About 5% to 10% of tuberculosis infections will progress to active TB disease in their lifetime, with an 80% risk in the first five years and a 50% risk in the first two years [6,7].

The tuberculin skin test is a crucial medical tool used to determine whether an individual has been infected with MTB. This test involves injecting tuberculin under the skin to assess the immune system’s response to the pathogen. The results of the skin test help identify whether an individual has been infected with MTB and the extent of the infection. For those in contact with patients with TB or at risk of contracting TB, the tuberculin skin test is a vital means of an early diagnosis and prevention of transmission. By detecting infections early on, healthcare professionals can implement appropriate interventions, including treatment and monitoring, to reduce the risk of TB spread, safeguarding the health of individuals and communities.

In 2014, the World Health Organization (WHO) defined the “End TB Strategy”, aimed at ending the global TB epidemic by 2035 [8]. As the third-highest-burden country for TB worldwide, China responded actively and implemented a national strategy. The “Healthy China 2030” blueprint explicitly stated that a comprehensive service model for TB prevention and control should be established [9]. This involves strengthening TB screening and monitoring and establishing a model that combines centralized hospitalization (CH) treatment care with a focus on a “patient-centered” approach. However, there was still a large proportion of patients with TB continuing to receive regular home-based (HB) management. In accordance with this guidance, we conducted the current study with two components: (1) a retrospective examination of medical records with the objective to compare the treatment outcome of CH and HB patients with tuberculosis who had finished their treatment; (2) a cross-section survey with an objective to compare TB infection among the household contacts of active patients with TB undergoing CH and HB treatment in Guizhou, China, covering the period from January 2022 to August 2023.

## 2. Methods

### 2.1. Study Setting

This study was based at four hospitals in Guizhou Province of China. Qingzhen People’s Hospital and Huangping County People’s Hospital provided CH care, while Sansui County People’s Hospital and Xishui County People’s Hospital provided HB treatment. All these hospitals have directly observed treatment (DOT) clinics for the management of patients with TB. 

For the CH group, the patients were admitted to the hospital to have anti-TB medication in the intensive phase for at least one month. During hospitalization, the patients and the relatives were also provided with health education related to TB and the importance of treatment adherence.

For the HB group, the patients were treated at home in a conventional way. Directly Observed Therapy was given by the relative at home, or if possible, at the health station.

After the two-month intensive phase, both groups of patients were treated at home based on the existing national guidelines. So, the two groups had the same treatment regimens. Their difference was only whether the patient was hospitalized or treated at home.

### 2.2. The Retrospective Analysis on Treatment Outcome

Data for the retrospective study is in the Supplement S1 file.

In this part, electronic data of all patients with TB retrieved from Guizhou Center for Disease Control (CDC) and from the study hospitals, spanning from January 2022 to August 2023, were analyzed. Three reviewers reviewed each record. 

The electronic data sets were retrieved from Guizhou CDC and the study hospitals. 

### 2.3. The Prospective Study

A cross-sectional survey was conducted on the household contacts of newly detected TB index patients. 

PTB index case: The patients who were recently diagnosed as patients with TB with sputum-smear-positive results for at least three sputum smear tests and registered in the National Tuberculosis Control Programme (NTP) during the study period.

Household contacts: The individuals who lived with the index cases for a minimum of 6 h per week over 2 months or longer or cohabited with the patients with index cases for more than a week before the index case was diagnosed as PTB.

The questionnaire used for data collection of this prospective study is in S2 file in the Supplement Materials.

### 2.4. Inclusion and Exclusion Criteria

For both studies, all patients consenting to CH and HB treatment received tuberculosis therapy before the commencement of this study. Patients with multidrug-resistant tuberculosis, those transferred out, and pregnant individuals requiring potential adjustments to standard tuberculosis drug regimens were excluded.

For the prospective study, the household contacts who tested positive for the chest X-ray and TST when the index case had finished and individuals with communication disorders were excluded.

### 2.5. Independent Variables and Outcome Variables for the Retrospective Part

The retrospective review of medical records of newly diagnosed PTB index cases was aimed at data collection to retrieve patient information for independent variables, especially age, gender, and the time (year) when treatment was given. Disease-specific parameters were also retrieved, including the results of a sputum acid-fast bacilli (AFB) smear. The treatment outcome was clarified, as shown in Table 1. The primary outcome of interest of the retrospective study was successful treatment.

Although the main comparison was to be focused on treatment outcomes, it is well known that these treatment outcomes are controlled by adherence to medication. For this reason, in the retrospective study, we first checked a possible mediating role of adherence to medication. Here, we conducted Baron and Kenny’s mediation analysis, putting adherence to medication as the mediatory factor. This means we first checked the association between the type of intervention (CH vs. HB), between the level of adherence to medication and, finally, between level of adherence to medication and treatment outcome. If adherence is a mediator, we should find that both steps of associations were statistically significant. Finally, we ran a regression model, including both types of intervention and the level of adherence simultaneously to predict the treatment outcome. The effect of the type of intervention in this regression would be its direct effect. All these analyses had confounders adjusted for. 

**Figure 1:**
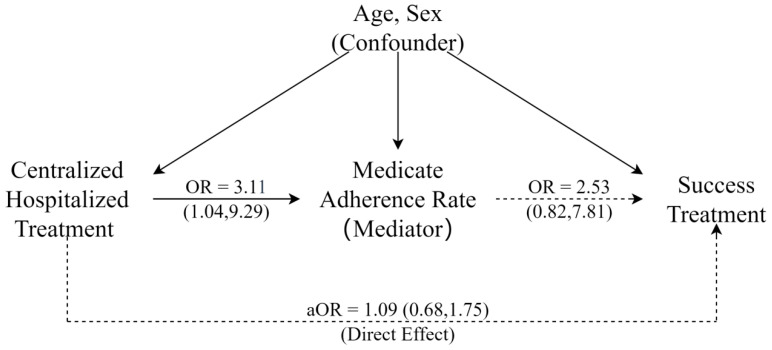
The relationship between the mode of treatment and outcome.

Figure 1 utilized a directed acyclic graph (DAG); we visually depicted the hypothesized causal pathways among the variables of interest, with centralized hospitalized treatment as the exposure variable directly impacting treatment success. Additionally, we conducted a mediation analysis to investigate the medication adherence rate as a potential mediator in this relationship. By systematically addressing confounding factors and considering mediation pathways, our aim was to offer a more comprehensive understanding of the relationship between centralized hospitalized treatment and treatment success, highlighting the potential roles of age, sex, and the medication adherence rate in this context.

The definitions are in accordance with the WHO TB treatment guidelines and NTP treatment outcomes [8,10]. The transferred outpatients were only captured in the review and the documentation, and were not evaluated as a treatment outcome and were excluded from the analysis of unsuccessful treatment.

The medication adherence, acting as a mediator variable, aims to verify whether hospitalization increases medication adherence and thereby enhances the treatment success rate.

### 2.6. Independent and Dependent Variables of the Prospective Study

The prospective cross-sectional survey consisted of closed-ended questions, with the questionnaire containing two sections. Section A included socio-demographic characteristics such as age, gender, ethnicity, educational qualification, occupation, marital status, the main breadwinner of the family, and whether to participate in CH. Section B contained clinical characteristics including results for TB screening, whether they received the Bacillus Calmette–Guérin (BCG) vaccine, the degree of closeness of contact with patients, and symptoms experienced at the onset of TB infection. 

The questionnaire and data collection form were designed by the investigators following extensive review of relevant studies [11,12,13,14,15,16,17,18], as well as based on previous practice experience. 

### 2.7. Sample Size Determination

The WHO sample size calculation for health studies was used to calculate the required sample size.

For Objective 1, the sample size calculation aimed to compare treatment outcomes between patients with TB with and without CH. The success rate of treatment for patients in CH was 93%, which is 5% higher than patients receiving treatment at home. Using the two independent proportion formulae with a continuity correction and assuming a 95% confidence level (α = 0.05), a power of 0.8 (β = 0.2), and equal sample sizes for both groups (r = 1), the minimum sample size for each group was determined as n_1_ = 539 and n_2_ = 539. The planned total sample size for recruitment was set at 1078 subjects. 

For Objective 2, the sample size calculation aimed to compare the proportions of developing tuberculosis infection and/or active PTB between household contacts of those two groups of patients with TB. The hypothesis of this study posits that among household contacts, close contacts regarding index cases, and individuals undergoing the TST, 10% are positive. The probability of household members contracting TB among isolated patients is expected to be two times lower than in the home treatment group. Using the two independent proportion formulae with a continuity correction and assuming a 95% confidence level (α = 0.05), a power of 0.8 (β = 0.2), and equal sample sizes for both groups (r = 1), the minimum sample size for each group was determined as n_1_ = 435 and n_2_ = 435. Considering a 10% non-response rate, the final planned total sample size for recruitment was set at 948 subjects. 

### 2.8. Data Collection for Household Contact Investigation

Essential training was provided to the local medical teams in each county and village involved in the research, including how to proficiently conduct questionnaire surveys. 

After receiving consent for home visits from the patients at the service station, at the visited household, the investigating members established contact with household members through village doctors. The investigators explained the significance of tracking household members and emphasized the advantages of participating in this study, which included health checks. Then, after the household members signed the informed consent forms, the investigators conducted questionnaire interviews with the household members. If any household members were children, informed consent from their guardians was obtained before TB screening. Even if household members declined participation in the questionnaire surveys, they might have still undergone TB screening by medical professionals. However, the data from these individuals were excluded from this study.

The screenings encompassed symptom assessment; results for the tuberculin skin test, X-rays, and sputum smear tests; and other relevant parameters.

Detecting the new infection cases among household contacts is significant in our study. All TSTs were performed at locally designed tuberculosis hospitals, with injecting five tuberculin units. The measurement of skin induration was documented 72 h after injection. The cutoff for considering a positive result was determined as ≥ 10 mm for the household contacts.

### 2.9. Data Analysis

All data were analyzed using R software (version 3.6.3). Descriptive statistics, such as frequencies and percentages, were used to summarize data for both prospective and retrospective cohorts. Treatment outcomes were evaluated for the retrospective cohort, including a successful treatment outcome (cured + treatment completed) versus failed treatment outcome (lost to follow-up + failed treatment + death by other causes + death by TB). The TST results of household contacts were evaluated for the prospective cohort. We employed the chi-square test for these variables. The significance level was set at *p* < 0.05. Univariate logistic regression was conducted for two objectives, and crude odds ratios were given along with 95% confidence intervals (CIs) and *p* values. In the multivariable logistic regression models for two objectives, all independent variables, and adjusted odds ratios, along with 95% CIs, were used to ascertain the degree of association between the dependent and independent variables, and a *p* value < 0.05 is deemed to be statistically significant.

### 2.10. Ethical Approval

This study was conducted in accordance with the Declaration of Helsinki and approved by the Ethics Committee of the Guizhou Provincial Center for Disease Control and Prevention (Approval No.: Q2023-13 dated 23 July 2023). It complies with the “Ethical Review Measures for Biomedical Research Involving Human Beings”, the “Management Measures for Medical Science and Technology Research Involving Human Subjects” (Draft for Solicitation of Opinions), and the Helsinki Declaration, among other relevant regulations.

## 3. Results

### 3.1. Retrospective Study 

#### 3.1.1. Characteristics of Participants

In the retrospective study, a total of 468 patients underwent CH, while 630 opted for home-based treatment. Table 2 outlines the characteristics of patients receiving CH and HB treatment based on the TB registration from 2022 to 2023. Of the participants, 468 patients underwent CH, whereas 630 opted for home-based treatment. These data highlight variations in patient demographics and medication adherence between the two treatment modalities (Table 2).

#### 3.1.2. Treatment Outcomes between Patients with TB with and without CH

Table 3 presents the treatment outcomes of patients with TB from the TB registration of the subjects described in Table 1. The success rate (cured + treatment completed) was 93.4% in the CH and 91.7% in the HB treatment group during the period. Adverse reactions and transfers to MDR therapy were minimal at 0.3% each. In summary, both treatment approaches demonstrated favorable outcomes, with a slightly higher cure rate in the CH group, while HB treatment showed a marginally higher completion rate. The incidence of defaults and treatment failure was relatively low in both groups, and the outcome of treatment was not significantly different (*p* value = 0.12) (Table 3).

Table 4 shows that the logistic regression analysis indicates a significant association between the medication adherence rate and the type of TB treatment after adjusting for confounding variables in the TB registration. The CH group had more than three times higher odds of having a ≥90% medication adherence rate (OR = 3.11, 95% CI: 1.04, 9.29). 

Table 5 displays the logistic regression results predicting treatment success, with the main variables (treatment mode, age, gender) included. Model 1 consists of the medication adherence rate as a covariate in the adjustment, but Model 2 does not. The odds ratios of CH in Model 2 reflect the total effect, whereas those in Model 1 indicate the direct effect bypassing the role of the medication adherence rate.

Figure 1 illustrates the relationship between the mode of treatment and outcome. Despite a high success rate in CH treatment, there was neither a direct effect nor an effect mediated through medication adherence on success treatment.

### 3.2. Prospective Study 

All participants enrolled in both hospitals during the study period provided consent to participate, resulting in a 100% response rate.

#### 3.2.1. Socio-Demographic Characteristics of Index Cases and Participants

Table 6 summarizes the characteristics of index cases of the CH and HB groups in the prospective study. Nearly two thirds of the index cases were on HB treatment. Most variables were well balanced, except the HB group was more likely to be Han than the CH group.

#### 3.2.2. Socio-Demographic and Clinical Characteristics of PTB Index Household Contacts

Table 7 illustrates the characteristics of the household contacts. For occupations, the highest proportion in the CH group was unemployed whereas that in the HB group was skilled worker. For ethnicity, the HB group was more likely to be Han, while the CH group was more inclined to have received the BCG vaccine. Both groups displayed a similar attendance rate for TB screening. The results of the TST did not differ significantly among the tested subjects in this crude analysis.

#### 3.2.3. Effect of CH Treatment on TST Result among the Household Contacts with Adjustment for Confounders

Table 8 summarizes the logistic regression results predicting the TST negativity among the two groups of contacts who underwent the TST. The effect of the treatment mode, initially non-significant in the univariate analysis, became significant in the logistic regression analysis after adjustment for confounders; the HB group had 26% odds of having a negative TST result compared to the CH group (aOR = 0.26, 95% CI: 0.07, 0.92). Females were 4.76 times more likely to have a negative TST result than males (aOR = 4.76, 95% CI: 1.23, 18.44). In terms of intensity of exposure to the patients with index TB, those who slept in the same room with the patient for more than two months had 2% odds of receiving a negative TST result compared to those who did not (aOR = 0.02, 95% CI: 0.01, 0.11).

## 4. Discussion

This study has brought to light that CH and HB therapy demonstrated comparable treatment success rates, standing at 94.8% and 93.1%, respectively. CH treatment resulted in better medication adherence, but neither a direct nor indirect effect of the treatment significantly increased the success rate. Moreover, household contacts within the CH group exhibited a 26% lower likelihood of having a positive TST result compared to their counterparts in the HB group. 

In the realm of pulmonary tuberculosis treatment success rates, Guangzhou achieved a notable 88% success rate (95% CI: 87–89%) from 1993 to 2002 [19]. In contrast, Hunan exhibited a treatment success rate of 93.1% between 2005 and 2006 [20], and in 2017, Ethiopia reached a treatment success rate of 91.9% [21]. When compared to Guangzhou, Hunan, and Ethiopia, Guizhou stands out for its proactive performance in pulmonary tuberculosis treatment. Particularly noteworthy is the significantly higher treatment success rate of 94.8% in Guizhou when employing CH therapy, surpassing the WHO target threshold of 90% [22]. This indicates that Guizhou has made progress in the treatment of pulmonary tuberculosis, and the use of CH may influence the treatment success rate, providing valuable information for the formulation of more effective prevention and intervention strategies.

Medication adherence is crucial for the effective management of tuberculosis in patients [23]. Medication adherence directly correlates with whether patients follow their prescribed medication regimen, thereby influencing the maintenance of drug concentrations at effective levels within the body. Consistently adhering to the prescribed medication regimen over the long term can effectively prevent the development of drug-resistant strains of MTB, ensuring the smooth progression of the treatment course [24,25]. CH may play a pivotal role in enhancing medication adherence. They have the potential to offer more convenient and patient-centered medical services, making it easier for patients to access and adhere to their treatment plans. Medical professionals in CH settings may have the capacity to comprehensively address individual differences among patients, providing more personalized medical advice. Furthermore, CH environments may foster closer relationships between patients and healthcare providers, encouraging patients to actively participate in the treatment process and thereby improving medication adherence. However, in our data, despite patients with over 90% adherence having 2.53 times higher odds of treatment success than those below 90%, we did not reach statistical significance. This was probably due to our limited sample sizes, with non-success rates of 5.3% and 6.9% in the CH and HB groups.

Household contacts in the HB group were found to only have a 26% likelihood of negative skin tests for tuberculosis compared to the CH group (aOR = 0.26, 95% CI: 0.07, 0.92). This finding underscores the potentially significant impact of isolating patients in hospitals on reducing the risk of tuberculosis infection among household contacts. Specifically, individuals with shared sleeping quarters for more than two months with the index case experienced a decrease in the likelihood of testing negative for tuberculosis (aOR = 0.02, 95% CI: 0.01, 0.11). Therefore, isolating patients in hospitals may be an effective measure to minimize close contact between patients and household contacts, thereby slowing the spread of the MTB and reducing the risk of infection among household contacts. The results are like previous studies, with the majority of household contacts [65.7% (115/175)] living in single-room houses. Among them, the highest prevalence of illness is observed in patients’ spouses [70.0% (14/20)] [26]. These research findings provide robust evidence supporting the implementation of isolation measures in tuberculosis management to ensure the health and safety of communities and households.

The success of CH therapy in improving adherence and reducing household infections underscores the potential of community health approaches in tuberculosis prevention. Policymakers and healthcare providers are advised to prioritize CH therapy, enhance medication adherence, and promote isolation measures for more effective and targeted interventions.

The management of elderly patients with comorbidities often benefits from a community setting or family support. However, the effectiveness of this approach varies significantly depending on the social, economic, and healthcare system conditions within a country. In contexts where community resources are robust and families can provide substantial support, this model can enhance patient well-being and reduce healthcare costs. Conversely, in areas with limited community infrastructure or strained familial networks, alternative strategies may be necessary to ensure optimal care for elderly individuals with multiple health concerns [27].

### Limitations

Information on medication adherence, although retrieved from the medical record, relies on participants’ unverified reports. This could be biased due to social desirability and memory errors.

The design of this study was not a randomized controlled trial. Subjects opting for CH and HB might already have differences in unrevealed aspects of backgrounds, which might influence the outcome. Influences of unknown confounders are unavoidable.

While the TST has been a longstanding and valuable tool in identifying individuals with latent tuberculosis, it is essential to consider certain limitations, such as cross-reactivity with the Bacillus Calmette–Guérin (BCG) vaccine and potential false-positive results in populations with previous exposure to non-MTB [28,29]. The test was conducted only on 20% of the contacts. Therefore, the information should be interpreted with caution.

## 5. Conclusions

Although there is no compelling evidence to suggest that CH for the treatment of TB yields higher success rates when compared to HB treatment, CH may increase medication adherence and may reduce the risk of transmitting tuberculosis infection and active PTB to household contacts. These positive effects suggest that CH policy should be continued in the study area.

## Figures and Tables

**Table 1:** Treatment outcomes for the PTB case in the retrospective study.

Outcome	Definition
Cured	Patients with TB with negative AFB smear or culture in the last month of treatment and on at least one previous occasion
Completed treatment	Patients with TB who completed treatment without evidence of failure but with no record of negative AFB smear or culture in the last month of treatment and on at least one previous occasion
Successful treatment	The sum of patients with TB who were cured and those who completed treatment
Failed treatment	Patients with TB with a positive AFB smear or culture at Month 5 or later during treatment
Unsuccessful treatment	The sum of patients with TB with treatment default and failed treatment and who have died
Lost to follow-up	An interruption of TB treatment for 2 or more consecutive months
Died	Patients with TB who died for any reason before starting or during the course of treatment

**Table 2:** Relevant CH treatment and HB treatment patients’ characteristics from the 2022 to 2023 TB registration.

Variable	CH Treatment	HB Treatment	Chi-Square Test *p* Value
Total	468	630	
Age (year)			<0.01
0–34	173 (37.0)	144 (22.9)	
35–59	165 (35.3)	276 (43.8)	
≥60	130 (27.8)	210 (33.3)	
Gender			0.36
Male	301 (64.3)	423 (67.1)	
Female	167 (35.7)	207 (32.9)	
Medication Adherence Rate			0.02
<90%	4 (0.9)	19 (3)	
≥90%	464 (99.1)	611 (97)	

**Table 3:** Relevant CH treatment and HB treatment patients’ treatment outcomes from the 2022 to 2023 TB registration.

Variable	CH Treatment	HB Treatment	Chi-Square Test *p* Value
Cured	298 (63.7)	355 (56.3)	0.12
Treatment completed	139 (29.7)	223 (35.4)	
Lost to follow-up	4 (0.9)	3 (0.5)	
Failed treatment	16 (3.4)	24 (3.8)	
Death by other causes	11 (2.4)	23 (3.7)	
Death by TB	0 (0.0)	2 (0.3)	

**Table 4:** Logistic regression evaluating probability of ≥90% medication adherence rate between CH and HB treatment with adjustment for confounding variable available among patients in the TB registration.

Variable	Medication Adherence Rate < 90%	Medication Adherence Rate ≥ 90%	Crude Odds Ratio	Adjust Odds Ratio	*p* Value from Likelihood Ratio Test
Treatment mode					0.024
HB treatment	19 (82.6)	611 (56.8)	1.00	1.00	
CH treatment	4 (17.4)	464 (43.2)	3.61 (1.22, 10.67)	3.12 (1.05, 9.32)	
Age (year)					0.019
0–34	2 (8.7)	315 (29.3)	1.00	1.00	
35–59	17 (73.9)	424 (39.4)	0.16 (0.04, 0.69)	0.22 (0.05, 1)	
≥60	4 (17.4)	336 (31.3)	0.53 (0.1, 2.93)	0.69 (0.12, 3.85)	
Gender					0.081
Male	20 (87)	704 (65.5)	1.00	1.00	
Female	3 (13)	371 (34.5)	3.51 (1.04, 11.9)	2.69 (0.78, 9.24)	

**Table 5:** Logistic regression models predicting treatment success, testing the effect of treatment mode with and without medication adherence rate included.

Independent Variable	Model 1	Model 2
Treatment mode		
Home-based treatment	1.00	1.00
CH treatment	1.09 (0.68, 1.75)	1.13 (0.71, 1.8)
Age (year)		
0–14	1.00	1.00
35–59	0.41 (0.19, 0.85)	0.39 (0.19, 0.81)
≥60	0.25 (0.12, 0.52)	0.25 (0.12, 0.52)
Gender		
Male	1.00	1.00
Female	0.92 (0.56, 1.49)	0.93 (0.57, 1.51)
Medication adherence rate		<Not included>
<90%	1.00	
≥90%	2.53 (0.82, 7.81)	

**Table 6:** Socio-demographics of PTB index patients.

Variable	CH Treatment, n (%)	HB Treatment, n (%)
Total	202 (36.9)	346 (63.1)
Age (year)		
0–19	10 (5.0)	12 (3.5)
20–49	74 (36.6)	125 (36.1)
50–64	54 (26.7)	95 (27.5)
≥65	64 (31.7)	114 (32.9)
Gender		
Male	131 (64.9)	226 (65.3)
Female	71 (35.1)	120 (34.7)
Educational level		
High school, vocational high school, or below	180 (89.1)	321 (92.8)
Universities, colleges, or above	22 (10.9)	25 (7.2)
Medical insurance		
Yes	200 (99)	337 (97.4)
None	2 (1)	9 (2.6)
Marital status		
Single	48 (23.8)	77 (22.3)
Married	148 (73.3)	246 (71.1)
Separated	0 (0.0)	2 (0.6)
Widowed	6 (3.0)	21 (6.1)
Occupation		
Student	16 (7.9)	18 (5.2)
Unemployed	76 (37.6)	121 (35.0)
Public/civil servant	5 (2.5)	7 (2.0)
Self-employed	53 (26.2)	96 (27.7)
Skilled worker	52 (25.7)	104 (30.1)
Ethnicity		
Han	110 (54.5)	257 (74.3)
Miao	83 (41.1)	46 (13.3)
Other ethnicity	9 (4.5)	43 (12.4)

**Table 7:** Socio-demographic and clinical characteristics of household contacts.

Variable	CH Treatment, n (%)	HB Treatment, n (%)	Chi-Square Test *p* Value
Total	559 (55.5)	448 (44.5)	
Age (year)			0.44
0–19	43 (7.7)	33 (7.4)	
20–49	271 (48.5)	218 (48.7)	
50–64	136 (24.3)	112 (25.0)	
≥65	109 (19.5)	85 (19.0)	
Gender			0.24
Male	264 (47.2)	218 (48.7)	
Female	295 (52.8)	230 (51.3)	
Educational level			0.57
High school, vocational high school, or below	511 (91.4)	415 (92.6)	
Universities, colleges, or above	48 (8.6)	33 (7.4)	
Medical insurance			0.46
Yes	558 (99.8)	443 (98.9)	
None	1 (0.2)	5 (1.1)	
Marital status			0.48
Single	118 (21.1)	77 (17.2)	
Married	429 (76.7)	360 (80.4)	
Separated	2 (0.4)	2 (0.4)	
Widowed	10 (1.8)	9 (2.0)	
Occupation			<0.01
Student	43 (7.7)	35 (7.8)	
Unemployed	234 (41.9)	138 (30.8)	
Public/civil servant	11 (2.0)	16 (3.6)	
Self-employed	146 (26.1)	104 (23.2)	
Skilled worker	125 (22.4)	155 (34.6)	
Ethnicity			<0.01
Han	366 (65.5)	339 (75.7)	
Miao	173 (30.9)	66 (14.7)	
Other ethnicity	20 (3.6)	43 (9.6)	
Main breadwinner of the family economy			0.01
Myself	131 (23.4)	112 (25.0)	
Family member	163 (29.2)	164 (36.6)	
Family members and myself	265 (47.4)	172 (38.4)	
BCG vaccine			<0.01
Yes	405 (72.5)	249 (55.6)	
No	154 (27.5)	199 (44.4)	
Relationship with the patient with TB			0.06
Sibling	76 (13.6)	31 (6.9)	
Husband/wife	132 (23.6)	121 (27.0)	
Parent	167 (29.9)	139 (31.0)	
Child	82 (14.7)	68 (15.2)	
Grandparent	29 (5.2)	21 (4.7)	
Others	73 (13.1)	68 (15.2)	
Intensity of exposure to patient with TB			
Living in the same house for more than two months	456 (81.6)	356 (79.5)	0.45
Eat at least one meal together every day for more than two months	216 (38.6)	172 (38.4)	0.99
Sleeping in the same room for more than two months	82 (14.7)	69 (15.4)	0.81
Responsible for caring for the patient for more than two months	132 (23.6)	139 (31.0)	0.01
Attended screening programs			
Chest X-ray	523 (93.6)	410 (91.5)	0.06
TST	105 (18.8)	100 (22.3)	0.11
Sputum AFB smear	122 (21.8)	96 (21.4)	0.94
Sputum culture	80 (14.3)	59 (13.2)	0.67
Results of the tuberculosis skin test (n = 205)			
Positive	16 (15.2)	26 (26)	0.06
Negative	89 (84.8)	74 (74)	

**Table 8:** Logistic regression analysis evaluates factors predicting TST negativity among TB household contacts.

Variable	Negative	Positive	Crude Odds Ratio	Adjust Odds Ratio	*p* Value from Likelihood Ratio Test
Has the patient been admitted to hospital *CH?					0.02
Yes	89	16	1.00	1.00	
No	74	26	0.51 (0.26, 1.03)	0.26 (0.07, 0.92)	
Age (year)					0.32
0–34	15	1	1.00	1.00	
35–64	33	11	0.18 (0.07, 0.5)	0.16 (0.02, 1.24)	
≥65	23	7	0.25 (0.07, 0.85)	0.14 (0.01, 1.60)	
Gender					0.02
Male	74	24	1.00	1.00	
Female	89	18	1.6 (0.81, 3.18)	4.76 (1.23, 18.44)	
Educational level					0.90
High school, vocational high school, or below	144	39	1.00	1.00	
Universities, colleges, or above	19	3	1.72 (0.48, 6.1)	0.64 (0.08, 5.22)	
Marital status					0.85
Married	120	27	1.00	1.00	
Single	42	14	0.67 (0.32, 1.41)	0.21 (0.03, 1.24)	
Others	1	1	0.23 (0.01, 3.71)	0.87 (0.02, 34.94)	
Occupation					0.55
Public/civil servant	6	2	1.00	1.00	
Unemployed	54	9	2 (0.35, 11.5)	0.74 (0.03, 19.74)	
Student	15	1	5 (0.38, 66.01)	0.63 (0.01, 76.92)	
Self-employed	32	7	1.52 (0.25, 9.19)	0.44 (0.02, 12.25)	
Skilled worker	56	23	0.81 (0.15, 4.32)	0.22 (0.01, 5.00)	
Main breadwinner of the family					0.06
Family member	53	7	1.00	1.00	
Myself	50	31	0.21 (0.09, 0.53)	0.34 (0.06, 1.84)	
Family members and myself	60	4	1.98 (0.55, 7.15)	8.73 (0.86, 88.20)	
Relationship with the patient with TB					0.90
Husband/wife	37	11	1.00	1.00	
Parent	48	11	1.3 (0.51, 3.32)	0.92 (0.14, 5.90)	
Others	78	20	1.16 (0.5, 2.67)	0.68 (0.14, 3.29)	
Intensity of exposure to patient with TB					
Living in the same house for more than two months	136	41	0.12 (0.02, 0.93)	0.19 (0.02, 1.99)	0.12
Living in the same house for less than two months	27	1	1.00	1.00	
Eat at least one meal together every day for more than two months	57	34	0.13 (0.05, 0.29)	0.71 (0.13, 3.90)	0.51
Eat at least one meal together every day for less than two months	106	8	1.00	1.00	
Sleeping in the same room for more than two months	22	33	0.04 (0.02, 0.1)	0.02 (0.01, 0.11)	<0.01
Sleeping in the same room for less than two months	141	9	1.00	1.00	
Responsible for caring for the patient for more than two months	38	28	0.15 (0.07, 0.32)	0.52 (0.12, 2.29)	0.38
Responsible for caring for the patient for less than two months	125	14	1.00	1.00	

## Data Availability

Requests to access these data should be sent to the corresponding author. The data sets used and/or analyzed during the current study are available from the corresponding author on reasonable request.

## Supplementary Materials

The following supporting information can be downloaded at: https://www.mdpi.com/article/10.3390/tropicalmed9050119/s1, File S1: Medical records of PTB index cases for the retrospective review. File S2: Questionnaire for the prospective study.
