# Supplementary material for: Evaluating Treatment Outcomes and Tuberculosis Infection Risks: A Comparative Study of Centralized Hospitalization vs. Home-Based Treatment
Source: Trop Med Infect Dis. 2024 May 18;9(5):119. doi: 10.3390/tropicalmed9050119 (PMC11125710; doi:10.3390/tropicalmed9050119)
Supplement: Supplementary file 1 [file tropicalmed-09-00119-s001.zip › Questionnaire for the prospective cross sectional study (chinese).docx]

**贵州省结核病集中和非集中住院治疗的效果及家庭接触者传播的风险的问卷调查**

**（非集中住院治疗）**

# 附件E.人口特征（非集中住院患者的基本情况）

| **编号** | **问 题** | **答 案** |
| --- | --- | --- |
| A1 | 号码（住院号） | [ ] [ ] [ ] [ ] |
| A2 | 年龄(不知道写888，不想回答写999) | [ ] [ ] [ ] |
| A3 | 性别 | [ ] 1. 男性  [ ] 2. 女性  [ ] 99. 拒绝回答 |
| A4 | 种族 | [ ] 1. 汉族  [ ] 2. 苗族  [ ] 3. 布依族  [ ] 4. 仡佬族  [ ] 5. 侗族  [ ] 6. 彝族  [ ] 7. 回族  [ ] 8. 土家族  [ ] 9. 水族  [ ] 10. 其他民族  [ ] 88. 不知道  [ ] 99. 拒绝回答 |
| A6 | 你家有几口人? | [ ] [ ] |
| A6 | 居住地址 | [ ] 1. 当地的（具体县名： ）  [ ] 2. 省内  [ ] 3. 跨省区  [ ] 99. 拒绝回答 |
| A7 | 你什么时候因肺结核住院的? | [ ] 1. 6月.2022  [ ] 2. 7月.2022  [ ] 3. 8月.2022  [ ] 4. 9月.2022  [ ] 5. 10月.2022  [ ] 6. 11月.2022  [ ] 7. 12月.2022  [ ] 8. 1月.2023  [ ] 9. [2月.](javascript:;)2023  [ ] 10. [三月.](javascript:;)2023  [ ] 88. 不知道  [ ] 9.拒绝回答 |
| A8 | 治疗持续（住院）时间(天) | [ ] [ ] [ ] |
| A9 | 登记类别 | [ ] 1. 新病人  [ ] 2. 复发  [ ] 3. 初始治疗失败  [ ] 4. 慢性病人  [ ] 99. 拒绝回答 |
| A10 | 职业 | [ ] 1. 企事业单位职工  [ ] 2. 国家机关，党群组织、企事业单位干部  [ ] 3. 个体经营户  [ ] 4. 农民  [ ] 5. 外出务工  [ ] 6. 本县区打零工，无固定职业  [ ] 7. 学生  [ ] 8. 医务人员  [ ] 9. 已退休  [ ] 10. 其他  [ ] 99. 拒绝回答 |
| A11 | 您家庭经济主要承担者 | [ ] 1. 本人  [ ] 2. 家属  [ ] 3. 您与家属分担  [ ] 99. 拒绝回答 |

**附件 F. 临床特征**

| **编号** | **问 题** | **答 案** |
| --- | --- | --- |
| B1 | 治疗管理 | [ ] 1. 全程监督  [ ] 2. 强化阶段监督  [ ] 3. 自行用药  [ ] 99. 拒绝回答 |
| B2 | 结核病诊断结果 | [ ] 1. 病原学检查阴性  [ ] 2. 涂片阳性  [ ] 3. 肺外结核  [ ] 4. 只有培养阳性  [ ] 5. 只有分子生物学阳性  [ ] 6. 没有病因学结果  [ ] 7. 只有病理阳性  [ ] 88. 不确定  [ ] 99. 拒绝回答 |
| B3 | 严重病例 | [ ] 1.是的  [ ] 2.不是  [ ] 3. 我不知道  [ ] 99. 拒绝回答 |
| B4 | 结核病治疗史 | [ ] 1.是的  [ ] 2.不是  [ ] 3. 我不知道  [ ] 99. 拒绝回答 |
| B5 | 结核病治疗结果 | [ ] 1. 治愈  [ ] 2. 治疗完成  [ ] 3. 治疗失败  [ ] 4. 转向耐多药(MDR)  [ ] 5. 无法跟进  [ ] 6. 不良反应  [ ] 7. 死  [ ] 8. 转移  [ ] 88. 不知道  [ ] 99. 拒绝回答 |
| B6 | 治疗后痰涂转阴 | [ ] 1.是的  [ ] 2.不是  [ ] 3. 我不知道  [ ] 99. 拒绝回答 |
| B7 | 治疗后痰培养转阴 | [ ] 1.是的  [ ] 2.不是  [ ] 3. 我不知道  [ ] 99. 拒绝回答 |
| B8 | 治疗后痰涂转阴时间 | [ ] [ ] [ ] |
| B9 | 治疗后痰培养转阴时间 | [ ] [ ] [ ] |
| B10 | 你家里有多少人感染过潜伏性肺结核? | [ ] [ ] [ ] |
| B11 | 你们有多少人家庭感染活跃肺结核? | [ ] [ ] [ ] |

**附件 G. 家庭接触者**

| **编号** | **问 题** | **答 案** |
| --- | --- | --- |
| C1 | 性别 | [ ] 1. 男性  [ ] 2. 女性  [ ] 99. 拒绝回答 |
| C2 | 种族 | [ ] 1. 汉族  [ ] 2. 苗族  [ ] 3. 布依族  [ ] 4. 仡佬族  [ ] 5. 侗族  [ ] 6. 彝族  [ ] 7. 回族  [ ] 8. 土家族  [ ] 9. 水族  [ ] 10. 其他民族  [ ] 88. 不知道  [ ] 99. 拒绝回答 |
| C3 | BCG接种疫苗 | [ ] 1. 是的  [ ] 2. 没有  [ ] 99. 拒绝回答 |
| C4 | 你和病人的关系? | [ ] . 父母关系  [ ] 2. 其他.  [ ] 99. 拒绝回答 |
| C5 | 病人肺结核情况 | [ ] 1. 涂片阴性  [ ] 2. 涂片阳性  [ ] 88. 不知道  [ ] 99. 拒绝回答 |
| C6 | 接触度 | [ ] 1.无接触  [ ] 2. 亲密  [ ] 99. 拒绝回答 |
| C7 | 每周接触时间 | [ ] [ ] [ ] |

**附件 H. 社会认可（度）**

| **编号** | **问 题** | **答 案** |
| --- | --- | --- |
| D1 | 这种治疗改善你的病情了吗? | [ ] 1.是的  [ ] 2.不是  [ ] 3. 我不知道  [ ] 99. 拒绝回答 |
| D2 | 这种治疗是否发生在高度管制的人群中医院的环境? | [ ] 1.是的  [ ] 2.不是  [ ] 3. 我不知道  [ ] 99. 拒绝回答 |
| D3 | 这种治疗对患者的生活质量有影响吗? | [ ] 1.是的  [ ] 2.不是  [ ] 3. 我不知道  [ ] 99. 拒绝回答 |
| D4 | 是否会有治疗有没有负面的社会影响? | [ ] 1.是的  [ ] 2.不是  [ ] 3. 我不知道  [ ] 99. 拒绝回答 |
| D5 | 这种治疗是否适合不同年龄的患者 | [ ] 1.是的  [ ] 2.不是  [ ] 3. 我不知道  [ ] 99. 拒绝回答 |
| D6 | 治疗是否能提高医护人员的工作效率和满意度? | [ ] 1.是的  [ ] 2.不是  [ ] 3. 我不知道  [ ] 99. 拒绝回答 |
| D7 | 你觉得集中住院和居家隔离疗效(集中) | [ ] 1.是的  [ ] 2.不是  [ ] 3. 我不知道  [ ] 99. 拒绝回答 |
|  |  |  |
