# Supplementary material for: Evaluating Treatment Outcomes and Tuberculosis Infection Risks: A Comparative Study of Centralized Hospitalization vs. Home-Based Treatment
Source: Trop Med Infect Dis. 2024 May 18;9(5):119. doi: 10.3390/tropicalmed9050119 (PMC11125710; doi:10.3390/tropicalmed9050119)
Supplement: Supplementary file 1 [file tropicalmed-09-00119-s001.zip › Questionnaire for the prospective cross sectional study.docx]

**Treatment Outcomes of Tuberculosis Patients in Centralization and Non-centralization Hospitalization and Related Impact on Their Household Contacts in Guizhou, China**

# Section A. Demographic Characteristics.

| **No** | **Questions** | **Answers** |
| --- | --- | --- |
| A1 | Unique ID | [ ] [ ] [ ] [ ] |
| A2 | Age in years (If don’t know, write 888, if refuse to answer, write 999) | [ ] [ ] [ ] |
| A3 | Sex | [ ] 1. Male  [ ] 2. Female  [ ] 99. Refuse to answer |
| A4 | Ethnicity | [ ] 1. Han  [ ] 2. Miao  [ ] 3. Buyie  [ ] 4. Kelao  [ ] 5. Dong  [ ] 6. Yi  [ ] 7. Hui  [ ] 8. Tujia  [ ] 9. Shui  [ ] 10. Other ethnic groups  [ ] 88. Don’t know  [ ] 99. Refuse to answer |
| A6 | How many people are there in your family? | [ ] [ ] |
| A6 | Residential address | [ ] 1. Local  [ ] 2. Intra-provincial  [ ] 3. Inter-provincial  [ ] 99. Refuse to answer |
| A7 | When were you hospitalized for tuberculosis？ | [ ] 1. Jun.2022  [ ] 2. Jul.2022  [ ] 3. Aug.2022  [ ] 4. Sep.2022  [ ] 5. Oct.2022  [ ] 6. Nov.2022  [ ] 7. Dec.2022  [ ] 8. Jan.2023  [ ] 9. [Feb.](javascript:;)2023  [ ] 10. [Mar.](javascript:;)2023  [ ] 88. Don’t know / Not sure  [ ] 9. Refuse to answer |
| A8 | Duration of therapy in days | [ ] [ ] [ ] |
| A9 | Registration category | [ ] 1. New patient  [ ] 2. Relapse  [ ] 3. Return after default  [ ] 4. Initial treatment failed  [ ] 5. Chronic patient  [ ] 99. Refuse to answer |
| A10 | Occupations | [ ] 1. Employees of enterprises and institutions  [ ] 2. Cadres of state organs, party-mass organizations, enterprises and institutions  [ ]3. Self-employed households  [ ] 4. Farmers  [ ] 5. Go out to work  [ ] 6. Part-time job in the county district, no fixed occupation  [ ] 7. Students  [ ] 8. Medical staff  [ ] 9. I'm retired  [ ] 10. Others  [ ] 99. Refuse to answer |
| A11 | Your family's main economic undertaker | [ ] 1. Yourself  [ ] 2. Family members  [ ] 3. Yourself and family members  [ ] 99. Refuse to answer |

**Section B. Clinical Characteristics**

| **No** | **Questions** | **Answers** |
| --- | --- | --- |
| B1 | Treatment management | [ ] 1. Full process supervision  [ ] 2. Intensive phase supervision  [ ] 3. Self-administered medication  [ ] 99. Refuse to answer |
| B2 | TB diagnosis results | [ ] 1. Etiological examination negative  [ ] 2. Smear positive  [ ] 3. Extrapulmonary TB  [ ] 4. Only culture positive  [ ] 5. Only molecular biology positive  [ ] 6. No etiological results  [ ] 7. Only pathologically positive  [ ] 88. Not sure  [ ] 99. Refuse to answer |
| B3 | Severely ill | [ ] 1. Yes  [ ] 2. No  [ ] 3. I do not know  [ ] 99. Refuse to answer |
| B4 | History of TB treatment | [ ] 1. Yes  [ ] 2. No  [ ] 3. I do not know  [ ] 99. Refuse to answer |
| B5 | TB treatment outcomes | [ ] 1. Cure  [ ] 2. Treatment completed  [ ] 3. Treatment failed  [ ] 4. Switch to multi-drug resistant (MDR)  [ ] 5. Lost to follow-up  [ ] 6. Adverse reactions  [ ] 7. Died  [ ] 8. Transfer out  [ ] 88. Not sure  [ ] 99. Refuse to answer |
| B6 | Sputum smear conversion | [ ] 1. Yes  [ ] 2. No  [ ] 3. I do not know  [ ] 99. Refuse to answer |
| B7 | Culture conversion | [ ] 1. Yes  [ ] 2. No  [ ] 3. I do not know  [ ] 99. Refuse to answer |
| B8 | Time of sputum smear conversion in days | [ ] [ ] [ ] |
| B9 | Time of culture conversation in days | [ ] [ ] [ ] |
| B10 | How many people in your family have been infected with latent tuberculosis? | [ ] [ ] [ ] |
| B11 | How many people in your family are infected with active tuberculosis? | [ ] [ ] [ ] |

**Section C. Household contacts**

| **No** | **Questions** | **Answers** |
| --- | --- | --- |
| C1 | Unique ID | [ ] [ ] [ ] [ ] |
| C2 | Age in years (If don’t know, write 888, if refuse to answer, write 999) | [ ] [ ] [ ] |
| C3 | Sex | [ ] 1. Male  [ ] 2. Female  [ ] 99. Refuse to answer |
| C4 | Ethnicity | [ ] 1. Han  [ ] 2. Miao  [ ] 3. Buyie  [ ] 4. Kelao  [ ] 5. Dong  [ ] 6. Yi  [ ] 7. Hui  [ ] 8. Tujia  [ ] 9. Shui  [ ] 10. Other ethnic groups  [ ] 88. Don’t know  [ ] 99. Refuse to answer |
| C5 | BCG vaccinated | [ ] 1. Yes  [ ] 2. No  [ ] 99. Refuse to answer |
| C6 | Your relationship with the patient? | [ ] 1. Parent  [ ] 2. Relatives or other people.  [ ] 99. Refuse to answer |
| C7 | The patient's tuberculosis condition | [ ] 1. Smear negative  [ ] 2. Smear positive  [ ] 3. I do not know  [ ] 99. Refuse to answer |
| C8 | Degree of contact | [ ] 1. Not  [ ] 2. Close  [ ] 99. Refuse to answer |
| C9 | Contact hours per week | [ ] [ ] [ ] |

**Section D. Social Acceptance**

| **No** | **Questions** | **Answers** |
| --- | --- | --- |
| D1 | Did this treatment improve your condition? | [ ] 1. Yes  [ ] 2. No  [ ] 3. I do not know  [ ] 99. Refuse to answer |
| D2 | Does this treatment take place in the highly regulated environment of the hospital? | [ ] 1. Yes  [ ] 2. No  [ ] 3. I do not know  [ ] 99. Refuse to answer |
| D3 | Does this treatment have an impact on the quality of life of patients? | [ ] 1. Yes  [ ] 2. No  [ ] 3. I do not know  [ ] 99. Refuse to answer |
| D4 | Whether the treatment will have any negative social effects? | [ ] 1. Yes  [ ] 2. No  [ ] 3. I do not know  [ ] 99. Refuse to answer |
| D5 | Whether this treatment is suitable for patients of different ages | [ ] 1. Yes  [ ] 2. No  [ ] 3. I do not know  [ ] 99. Refuse to answer |
| D6 | Whether treatment can improve the efficiency and satisfaction of health care workers? | [ ] 1. Yes  [ ] 2. No  [ ] 3. I do not know  [ ] 99. Refuse to answer |
